# Evaluation of folate receptor-alpha and other surface markers as potential targets for radionuclide therapy of ovarian cancer

**DOI:** 10.1186/s13550-025-01345-0

**Published:** 2025-12-29

**Authors:** Benjamin D. Hunkeler, Jakob Heimer, Ana Katrina Mapanao, Matthias Choschzick, Cristina Müller, Niels J. Rupp

**Affiliations:** 1 Center for Radiopharmaceutical Sciences, PSI Center for Life Sciences, Villigen-PSI, Switzerland; 2 Digital Trial Innovation Platform, ETH Zurich, Zurich, Switzerland; 3 Department of Pathology and Molecular Pathology, University Hospital Zurich, Zurich, Switzerland; 4 Department of Chemistry and Applied Biosciences, ETH Zurich, Zurich, Switzerland; 5 Faculty of Medicine, University of Zurich, Zurich, Switzerland

**Keywords:** Ovarian cancer, Folate receptor-alpha, Fibroblast activation protein, Immunohistochemistry

## Abstract

**Background:**

Epithelial ovarian cancer (EOC) has the highest mortality rate among gynecological malignancies, primarily due to frequent late-stage diagnosis and the development of resistance to chemotherapy. The aim of this study was to evaluate the expression of tumor-associated targets in a large cohort (*n* = 179) of various EOC subtypes, represented on two tissue microarrays (TMAs), to support the future development of radionuclide therapies. The study primarily assessed folate receptor-alpha and -beta isoforms (FRα and FRβ), but also somatostatin receptor-2 (SSTR2), prostate-specific membrane antigen (PSMA) and fibroblast activation protein (FAP), for which established radiopharmaceuticals already exist. Membranous expression of these targets on tumor cells was detected by immunohistochemistry using antibodies validated on xenografts with known target expression and semi-quantitatively evaluated.

**Results:**

Validation of the employed antibodies confirmed specific staining of the respective targets. The TMAs included tumors of high-grade and low-grade serous, endometrioid, clear cell, mucinous and carcinosarcoma. High FRα expression was seen in several EOC histotypes, most frequently in high-grade serous (47%), while it was largely absent in mucinous EOC. The FRβ was expressed only in stromal cells. SSTR2 and PSMA were only present in 8% and 4% of the EOC cases and not associated to a specific subtype. FAP expression on tumor cells was found in 10% of all EOCs, while stromal FAP was seen in 47% of the cases, with the highest prevalence in high-grade serous EOC (42%).

**Conclusions:**

The findings of this study indicate that approved radionuclide therapies targeting SSTR2 or PSMA are unlikely to be suitable for treating EOC. In contrast, the frequent and high expression of FRα in tumor cells and FAP in tumor-associated stromal cells suggests that FRα- and FAP-targeted radiopharmaceuticals hold promise for the treatment of advanced-stage EOC.

**Supplementary Information:**

The online version contains supplementary material available at 10.1186/s13550-025-01345-0.

## Introduction

Epithelial ovarian cancer (EOC) accounts for approximately 90% of ovarian cancers and remains the gynecological malignancy with the highest mortality worldwide [1]. Due to non-specific symptoms, EOC is frequently diagnosed at an advanced stage, leading to an unfavorably low five-year survival rate of only 30% [2]. Therapeutic standard of care combines cytoreductive surgery with neoadjuvant or adjuvant platinum-based chemotherapy. While initial remission is frequently observed, up to 70% of patients relapse within 18‒28 months and often with platinum-resistant disease [3].

Tumor-targeted therapy using antibodies or small molecules conjugated to highly toxic payloads is a promising strategy to overcome the limitations of conventional chemotherapy and improve patient outcomes in recurrent and chemoresistant disease. The folate receptor-α (FRα), a cell membrane-anchored glycoprotein, was proposed as a relevant tumor-associated target in this regard [4]. Elevated FRα-expression levels were reported for ovarian, endometrial and breast cancer, among others [5, 6]. In normal tissue, its expression was only detected at a few sites, such as in the kidneys, lungs and choroid plexus of the brain [4]. The folate receptor-beta (FRβ) is another folate receptor isoform, which also binds folic acid with high affinity [7]. It has been found expressed on activated macrophages involved in inflammatory processes [8]. While there is limited evidence of FRβ expression on epithelial tumor cells [9], its presence was reported on stromal macrophages in EOC and other cancer types [10, 11].

Mirvetuximab soravtansine is a FRα-targeting monoclonal antibody-drug conjugate, which was approved by the United States Food and Drug Administration (FDA) in 2022 for the treatment of FRα-positive platinum-resistant ovarian cancer [12]. In the same year, the immunohistochemistry (IHC)-based companion diagnostic test FOLR1-2.1 received FDA approval as a means to identify patients eligible for therapy with mirvetuximab soravtansine. The FOLR1-2.1 test comes with the PS2+ scoring system, which combines the proportion of FRα-expressing tumor cells with a four-level staining intensity scale (0 to 3+). The PS2+ designates tumors as “FRα-high” if at least 75% of tumor cells exhibit moderate (2+) and/or strong (3+) membranous staining. This test facilitated the identification of patients who were likely to benefit from mirvetuximab soravtansine treatment, resulting in objective response rates of 32–42% in the SORAYA (NCT04296890) and MIRASOL (NCT04209855) Phase III trials [12, 13]. Despite its utility, an IHC-based assessment does not provide comprehensive information about inter- and intra-tumor heterogeneity of FRα expression, especially in metastatic disease. Furthermore, establishing reliable IHC-based cutoffs for selecting patients with FRα-high tumors has proven to be challenging [14, 15]. In this regard, the use of radiopharmaceuticals that can specifically bind to the FRα offer a reliable identification and visualization of disseminated FRα-positive tumor lesions through positron emission tomography (PET) and single-photon emission computed tomography (SPECT). Folic acid-based radiopharmaceuticals have, therefore, been developed to allow non-invasive imaging of FR-positive tumors, as well as the assessment of target expression, intratumoral heterogeneity and receptor accessibility across the entire metastatic burden [16–18].

In addition to folic acid-based nuclear imaging agents, the use of therapeutic radiopharmaceuticals, designed to selectively deliver therapeutic radiation to FRα-positive cancer cells, has been proposed and exemplified in preclinical studies [19]. While this approach has not been translated yet for patient use, targeted radionuclide therapy has been established in clinics using somatostatin analogues such as [^177^Lu]Lu-DOTATATE (Lutathera™) for targeting somatostatin receptor-2 (SSTR2)-positive neuroendocrine neoplasms [20]. More recently, [^177^Lu]Lu-PSMA-617 (Pluvicto™) that binds to the prostate-specific membrane antigen (PSMA) has been approved for the treatment of metastatic castration-resistant prostate cancer [21]. These therapeutic radiopharmaceuticals significantly changed the clinical management of patients suffering from metastasized diseases of the respective tumor entities [22]. Another tumor-associated protein that has gained attention as a theragnostic target is the fibroblast activation protein (FAP), which is often expressed in the stroma of epithelial cancers and on tumor cells of EOC [23, 24]. The high prevalence of FAP in epithelial tumors led to the development of multiple FAP-targeted PET imaging agents and, to a small extent, also therapeutic radiopharmaceuticals [25]. In order to extend the application of targeted radionuclide therapy to metastasized disease of EOCs, there is a need to evaluate potential tumor-associated targets.

The goal of this study was to evaluate the expression of FRα and FRβ in tumors obtained from treatment-naïve EOC patients and assess the potential of folate-based radiopharmaceuticals for radiotheragnostic interventions in EOC. Furthermore, we explored the expression patterns of SSTR2, PSMA and FAP in EOC to determine whether established radiopharmaceuticals could be employed for the treatment of EOC.

## Methods

### Antibody validation on mouse xenograft tissue

Cells of known target expression (human FRα/FRβ, human SSTR2, human PSMA and human FAP, respectively) were grown in mice to obtain xenografts and related xenograft paraffin sections (Supplementary Information). The studies were performed in accordance with the available licenses and ethical approval by the Swiss Cantonal Committee of Animal Experimentation (License: N° 75721 and 75668). Female severe combined immunodeficient (SCID) CB17 (CB17/lcr-*Prkdc*^*scid*^/lcrlcoCrl) mice, CD1 nude (Crl: CD1-Foxn^nu^) and BALB/c nude mice (Foxn1^nu^/Crl) were obtained from Charles River Laboratories (Sulzfeld, Germany). To confirm the specific binding of the monoclonal mouse anti-FRα antibody (26B3.F2; Biocare Medical, Pacheco, USA) and the polyclonal rabbit anti-FRβ antibody (GTX105822, GeneTex, Irvine, USA), xenografts were obtained from transfected Chinese hamster ovary (CHO) cells which exclusively express the FRα (CHO-FRα, RT16) or FRβ (CHO-FRβ, D4) [26]. These cell lines were kindly provided by Prof. Larry H. Matherly (Wayne State University, Detroit, USA) [27]. The polyclonal rabbit anti-SSTR2 antibody (RBK046-05, Zytomed Systems, Langenzersdorf, Austria) was validated using tumor xenografts of the human pancreatic neuroendocrine BON tumor cells and the SSTR2-transfected BON-SSTR2 cells, which were both kindly provided by Dr. Carsten Grötzinger (Charité Universitätsmedizin, Berlin, Germany) [28]. PSMA staining with the monoclonal mouse anti-PSMA antibody (3E6; DAKO A/S, Glostrup, Denmark) was validated using xenografts of the human prostate cancer cell lines PC-3 PIP (PSMA-positive) and PC-3 flu (PSMA-negative), which were kindly provided by Prof. Martin Pomper (Johns Hopkins University School of Medicine, Baltimore, USA). Validation of the monoclonal rabbit anti-FAP antibody (EPR20021; Abcam, Cambridge, UK) was performed using xenografts derived from the HT1080 human fibrosarcoma cell line and the respective FAP-transfected human fibrosarcoma cell line (HT1080-FAP), kindly provided by Prof. Christoph Renner (Hirslanden Clinics, Zurich, Switzerland) [29].

### Patient material and ethics statement

In this study, a cohort of *n* = 179 treatment-naïve patients diagnosed with EOC of various histotypes, including high-grade serous, low-grade serous, endometrioid, mucinous, clear cell, carcinosarcoma and mixed histotype, was investigated. From each of the 179 EOC tumors, 1‒2 tissue cores of 0.5 mm diameter had previously been obtained and the *n* = 358 tissue cores arranged on two tissue microarrays (TMAs). The histotyping of tissue cores was performed according to the latest World Health Organization (WHO) Classification of Female Genital Tumors (5th Edition) [30]. The specimens were collected between 1997 and 2008 at the University Hospital Zurich, Zurich, Switzerland. Written general consent was substituted by the ethics vote for inclusion of tissue and data from before 2016. Ethical approval was granted by the cantonal ethics committee Zurich, Switzerland (BASEC 2022 − 01778).

### Immunohistochemical staining of paraffin-embedded tissues

Tissue sections of 2 μm thickness were cut from formalin-fixed paraffin-embedded tissues for immunohistochemistry. Immunohistochemical staining of FRα, SSTR2 and PSMA was performed on the Ventana Benchmark automated staining system (Roche Diagnostics, Rotkreuz, Switzerland) and FRβ and FAP on the Leica BOND III Stainer (Leica Biosystems, Nussloch, Germany) at the Institute of Pathology and Molecular Pathology of the University Hospital Zurich, Zurich, Switzerland using the validated antibodies (see above; Supplementary Information; Table S1). Immunohistochemical staining of the FRα and FRβ was performed with the prediluted (ready-to-use) monoclonal mouse anti-FRα antibody and polyclonal rabbit anti-FRβ antibody (1:1600 dilution). Whole slide sections of a total of *n* = 4 tumors of high-grade serous, low-grade serous and endometrioid histotypes with FRα-high and FRβ-negative status were analyzed in addition to the TMAs to confirm the representability of the TMA cores of the respective tumor. The staining of the SSTR2 was performed using the polyclonal rabbit antibody RBK046-05 (1:25 dilution), while PSMA and FAP were stained using the monoclonal mouse antibody 3E6 (1:25 dilution) and the monoclonal rabbit antibody EPR20021 (1:100 dilution), respectively. The optiView DAB-kit (Roche Diagnostics, Rotkreuz, Switzerland) and the BOND DAB kit (Leica Biosystems, Nussloch, Germany) were employed for detection of antibody binding on the Ventana Benchmark and Leica BOND III stainer, respectively (Supplementary Information). The slides were scanned using a NanoZoomer scanner (Hamamatsu, Shizuoka, Japan) and evaluated using the viewing software NDP.view2 version 2.9.29 (Hamamatsu, Shizuoka, Japan). Due to loss of tissue on TMAs during immunohistochemical processing the total number of evaluable tumors varied across the investigations for the various targets: FRα (*n* = 160), FRβ (*n* = 167), SSTR2 (*n* = 164), PSMA (*n* = 164), tumor cell FAP (tFAP; *n* = 158) and stromal FAP (stFAP; *n* = 155).

### Evaluation strategy

**Fig. 1:**
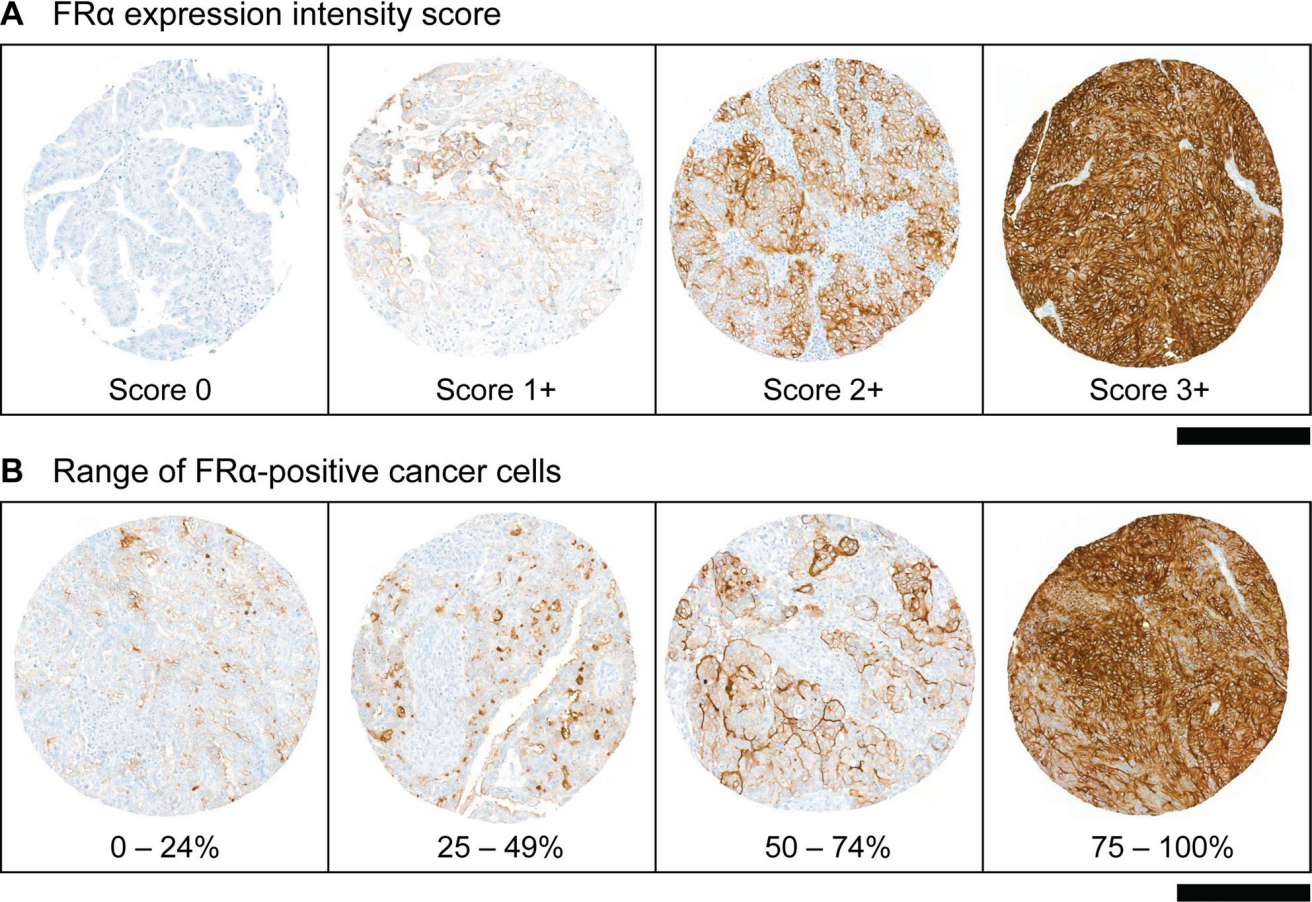
Evaluation of FRα expression shown with representative tissue microarray cores. **A** Qualitative expression intensity score (0 to 3+). **B** Range of percentage of FRα-positive cancer cells. (Scale bars: 250 μm)

Both cytoplasmic and membranous histochemical staining was observed; however, only membranous expression of the respective receptor was considered for the evaluation. Membranous immunohistochemical staining intensity was semi-quantitatively evaluated using a scoring system from 0 to 3+ (0 = negative, 1+ = weak, 2+ = moderate, 3+ = strong) (Fig. 1A) and the fraction of positive cells recorded in 1% increments up to a fraction of 5%, followed by 5% steps for fractions >5% (Fig. 1B). In heterogeneous cases, the most prominent pattern was analyzed. An immunoreactivity score (IRS) was obtained by multiplying the intensity score with the fraction of cancer cells with membrane staining (IRS = intensity [score] x fraction [%]). The obtained staining intensity scores and percentages obtained from the same tumor of a patient were averaged. FRα expression was considered as “FRα-high” if the staining intensity was 2+ and/or 3+ observed in ≥ 75% of tumor cells. This corresponded to an IRS ≥ 150, based on the PS2+ criteria applied in the SORAYA study (Table 1) [31]. “FRα-low” cases showed any expression lower than the FRα-high criteria, whereas cases that did not show any expression were classified as “FRα-negative”. Membranous FRβ, SSTR2, PSMA and tumor cell-associated FAP (tFAP) expression in epithelial tumor cells were analyzed in an analogous manner to the FRα, with a four-level expression score (0 to 3+) and percentage of positive tumor cells. Expression of FAP in the stroma (stFAP) was considered “stFAP-high” when more than 50% of the stroma displayed a staining intensity of ≥ 2+, whereas expression below this threshold was classified as “stFAP-low” and no expression as “stFAP-negative”.

**Table 1:** Criteria for evaluation of epithelial FRα, FRβ, SSTR2, PSMA and FAP expression adapted from James et al. [31]

Expression status	Immunoreactivity score (IRS)	Observation
High	IRS ≥ 150	≥ 75% of tumor cells with moderate (2+) or strong (3+) membrane staining
Low	0 < IRS ≤ 210	< 75% of tumor cells with moderate (2+) or strong (3+) membrane staining
Negative	IRS = 0	100% of tumor cells without (0) membrane staining

### Statistical analysis

Analyses were conducted using R statistical software (version 4.4.1), using the “emmeans” package for post-hoc comparisons. In cases where more than one core was evaluated, the respective scores and percentage of positive tumor cells were indicated as the arithmetic average of both samples. The association of FRα expression levels with the EOC histotype was modeled using patient-level logistic regression (binomial family, logit link), with histotype category as the predictor variable. “High-grade serous” served as the reference category for all histotype comparisons. From the fitted model, estimated marginal means (EMMs) on the log-odds scale and their corresponding 95% confidence intervals were derived for each histotype. Pairwise contrasts were performed to compare each non-reference histotype against the “High-grade serous” reference level; p-values were reported without adjustment for multiple comparisons. Statistical significance was established at *p* < 0.05.

## Results

### Antibody validation

**Fig. 2:**
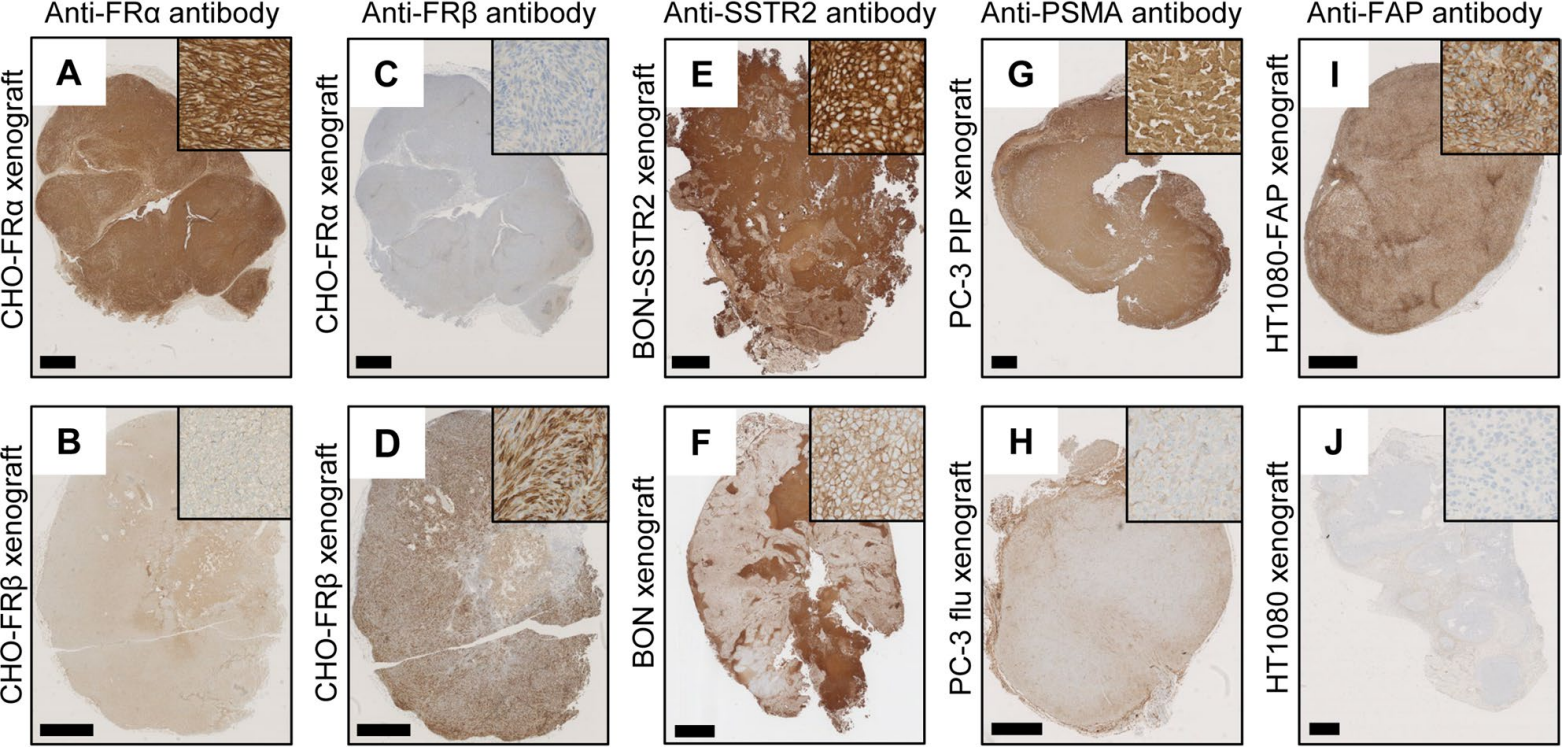
**A-J** Antibody validation on xenografts with high or no/low expression of the investigated targets. IHC staining of **A/B** FRα using anti-FRα and **C/D** FRβ using anti-FRβ antibodies on **A/C** CHO-FRα xenografts and **B/D** CHO-FRβ xenografts. IHC detection of SSTR2 on **E** BON-SSTR2 and **F** BON xenografts. IHC staining of PSMA on **G** PC-3 PIP and **H** PC-3 flu xenografts. Detection of FAP on xenografts of **I** transfected HT1080-FAP and **J** HT1080 cells. (Scale bars: 1 mm)

The specificity of the antibodies against FRα and FRβ was confirmed on paraffin tissue sections of CHO-FRα and CHO-FRβ xenografts, respectively. The immunohistochemistry demonstrated specific binding of the anti-FRα antibody to the CHO-FRα, while a faint signal with no clear membranous localization was observed on the CHO-FRβ xenograft (Fig. 2A/B). In contrast, the anti-FRβ antibody showed an opposite pattern with clear membranous staining in the CHO-FRβ xenograft and absence of signal in the CHO-FRα xenograft (Fig. 2C/D). SSTR2 was highly present in BON-SSTR2 xenograft sections and was low on sections of BON xenografts, consistent with the anticipated expression profiles (Fig. 2E/F). PSMA expression was visualized on sections of PSMA-positive PC-3 PIP xenografts, while no membranous signal was detected on PSMA-negative PC-3 flu xenograft sections (Fig. 2G/H). Similarly, the antibody against FAP demonstrated strong staining in xenografts derived from the FAP-transfected fibrosarcoma cell line HT1080-FAP while only low expression was seen in the wild-type cells of HT1080 xenografts (Fig. 2I/J).

### Patient and tumor characteristics

The most prevalent histotype investigated in this study was high-grade serous carcinoma (*n* = 86, 48% of total patients), followed by endometrioid (*n* = 38, 21%) and clear cell carcinoma (*n* = 22, 12%). The mucinous histotype was represented by *n* = 15 patients (8%) and carcinosarcoma by *n* = 9 (5%). The least frequent histotypes in the cohort were low-grade serous (*n* = 7, 4%) and mixed histotype carcinoma (*n* = 2, 1%) (Supplementary Information; Table S2).

### Folate receptor-alpha (FRα) and folate receptor-beta (FRβ) expression

**Fig. 3:**
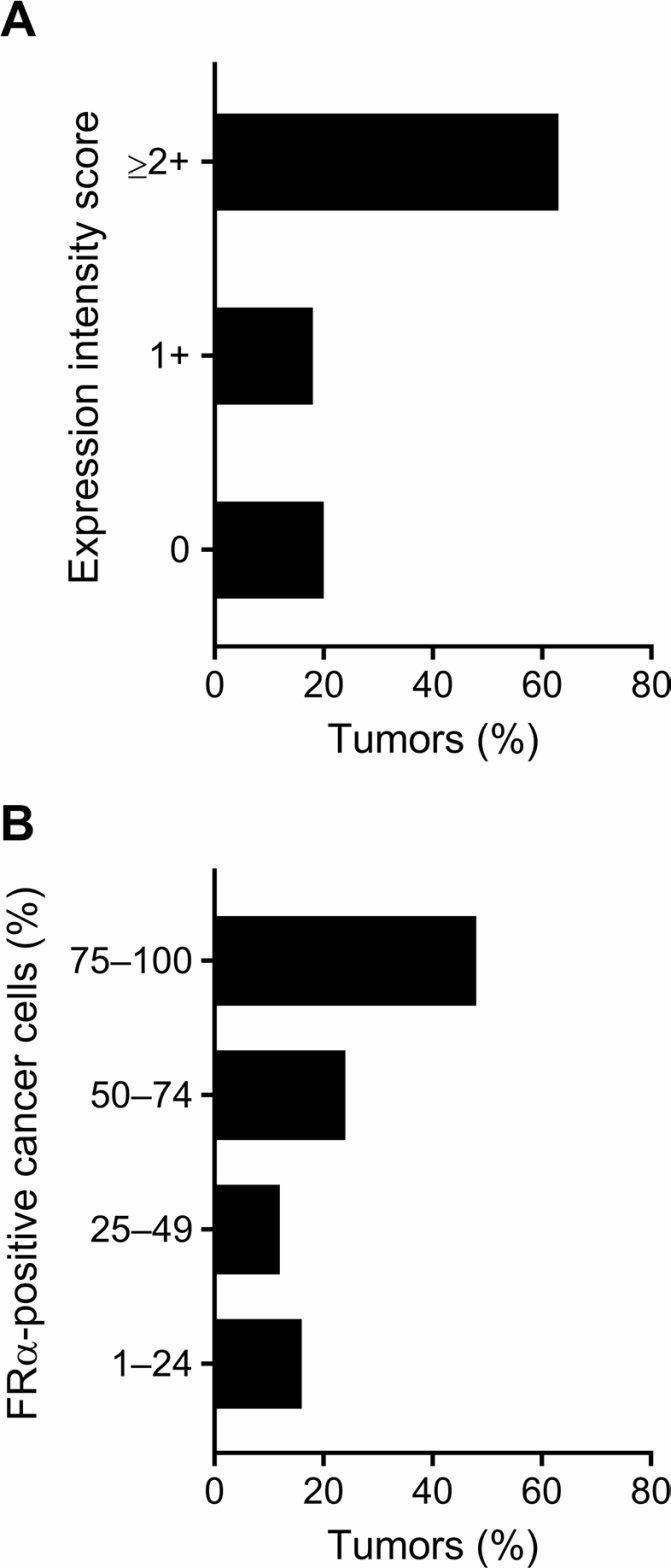
Immunohistochemical evaluation of the FRα expression in ovarian carcinoma tumors (*n* = 160) irrespective of the histotype. **A** Percentage of tumors showing no (0), low (1+) or moderate-to-high (≥ 2+) membranous FRα expression on tumor cells. **B** Percentage of tumors consisting of different proportions of FRα-positive tumor cells

In total, *n* = 160 tumors were evaluated for FRα expression and *n* = 19 tumors had to be excluded due to loss of TMA cores during the staining process. Membranous FRα expression of any intensity was observed in 80% (*n* = 128) of tumors with an average IRS of 130. Strong membrane staining intensity (≥ 2+) was observed in 63% of the tumors (*n* = 100). Low FRα expression was seen only in 18% (*n* = 28) of the cases while FRα was absent in 20% (*n* = 32) of the tumors (Fig. 3A). Among FRα-positive tumors, 48% of the cases (*n* = 61) showed a high fraction (75–100%) of FRα-positive tumor cells. The prevalence of FRα expression in the remaining tumor samples was lower, with 50–74% FRα-positive tumor cells in 24% of tumors (*n* = 31) and 1–49% FRα-positive tumor cells in 28% of cases (*n* = 36) (Fig. 3B).

**Fig. 4:**
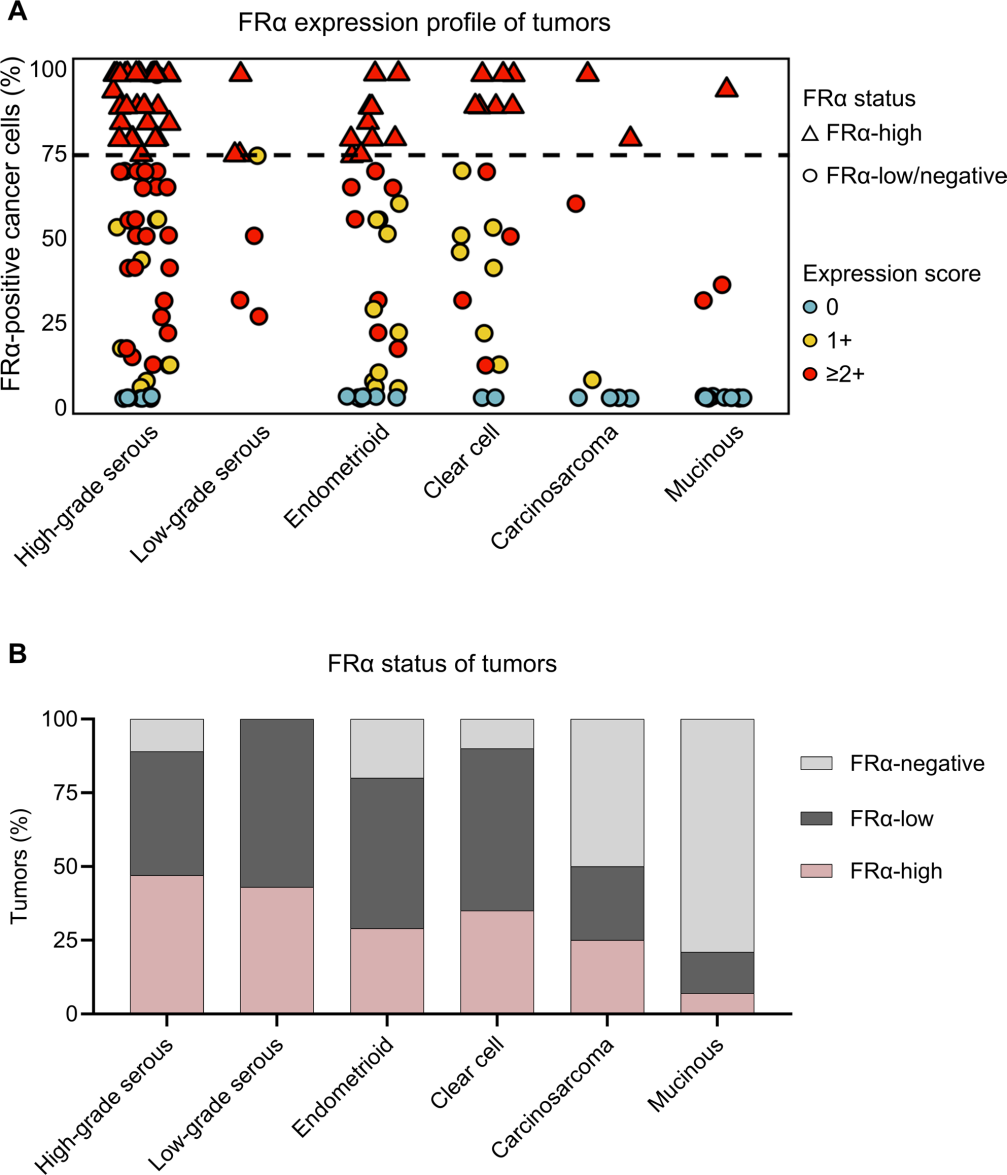
FRα status of ovarian carcinoma tumors. **A** FRα expression score and percentage of FRα-positive tumor cells observed in each tumor and the resultant FRα-status. Dashed line corresponds to 75% FRα-positive tumor cells set as threshold for FRα-high according to PS2+ scoring system. **B** Fraction of tumors scoring FRα-high, FRα-low or FRα-negative categorized by histotype

The expression was considered FRα-high if 75% of the tumor cells showed a staining intensity score of ≥ 2+. Based on this threshold, 37% (*n* = 59) of tumors were scored as FRα-high, while 43% (*n* = 69) tumors and 20% (*n* = 32) of tumors were identified as FRα-low and FRα-negative, respectively (Fig. 4). FRα-high cases were observed across different histotypes, with 47% prevalence in high-grade serous (*n* = 36) and 43% in low-grade serous carcinomas (*n* = 3). Frequencies of FRα-high status in endometrioid and clear cell carcinomas were comparable with 29% (*n* = 10) and 35% (*n* = 7), respectively. In the case of carcinosarcoma, FRα-high expression was found in 25% of the cases (*n* = 2), whereas only 7% (*n* = 1) was identified in the mucinous histotype (Supplementary Information; Table S3). In total, *n* = 4 tumors of high-grade and low-grade serous and endometrioid histotypes that were classified as FRα-high were selected for whole slide analysis of FRα expression. In agreement with the observation made on the respective TMA cores of these tumors, the whole slides displayed high FRα expression levels (Supplementary Information; Fig. S1).

A logistic regression analysis identified a significant difference in the frequency of tumors scoring FRα-high among different histotypes, with high-grade serous set as the reference (Table 2). Mucinous carcinomas demonstrated significantly lower odds of FRα-high positivity compared to the high-grade serous group (log(OR) = −2.5; *p* = 0.021). In the case of endometrioid subtype, the comparison revealed a trend towards lower odds which did not reach statistical significance (log(OR) = −0.81; *p* = 0.065), while the histotypes carcinosarcoma, clear cell and low-grade serous EOC did not significantly differ in FRα-high odds compared to high-grade serous EOC (*p* > 0.05).

**Table 2:** Comparison of FRα-high frequencies among ovarian carcinoma histotypes

Category	log(OR)^a^	95% CI^a^	*p*-value^b^
High-grade serous	—	—	—
Low-grade serous	−0.18	−1.9, 1.4	0.82
Endometrioid	−0.81	−1.7, 0.03	0.065
Clear cell	−0.51	−1.6, 0.49	0.33
Carcinosarcoma	−0.99	−3.0, 0.55	0.24
Mucinous	−2.5	−5.4, −0.77	0.021
^a^ OR = Odds Ratio, CI = Confidence Interval ^b^ Determined by logistic regression analysis			

No membranous epithelial FRβ expression was observed in *n* = 167 evaluated tumors. The staining of tissue on whole slides (*n* = 4) showed heterogeneous, faint cytoplasmic FRβ expression without membranous localization in the tumor cells, whereas some cells within the stroma showed membranous FRβ expression (Supplementary Information; Fig. S2).

### Somatostatin receptor-2 (SSTR2) expression

**Fig. 5:**
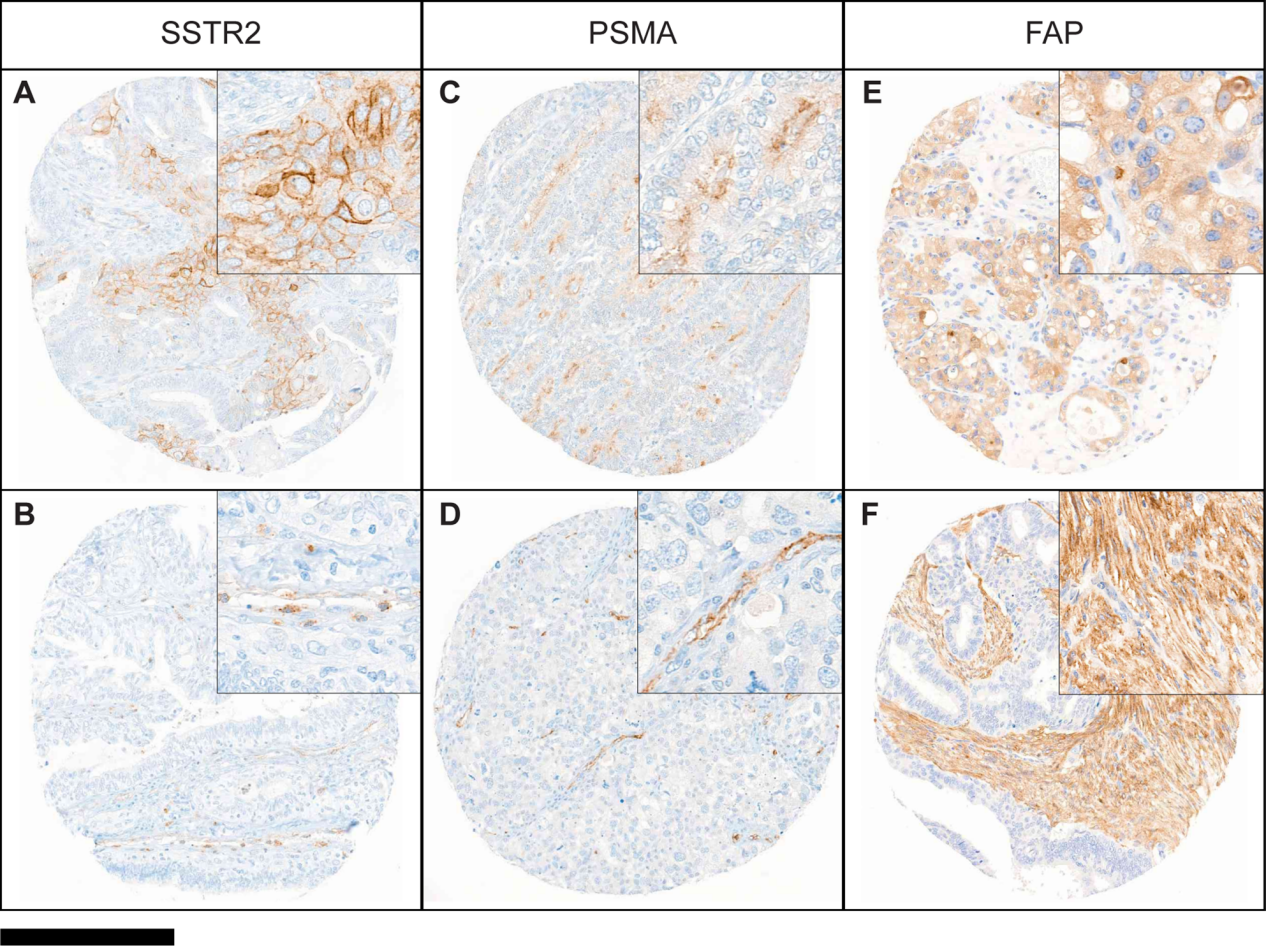
**A-F** Representative tissue cores with expression of SSTR2, PSMA and FAP on tumor cells, endothelial cells and stromal cells. **A/B** SSTR2 expression; **C/D** PSMA expression; **E/F** FAP expression. **A/C/E** Staining of tumor cells; **B/D** endothelial cells and **F** stromal cells. (Scale bar: 250 μm)

Expression of SSTR2 was assessed in *n* = 164 tumors. Membranous SSTR2 expression was noted in 8% of the tumors (*n* = 13) (Fig. 5A). Among the SSTR2-positive tumors, 6% (*n* = 10) displayed low SSTR2 expression with an IRS < 50, whereas the remaining tumors (*n* = 3) exhibited higher SSTR2 expression with an IRS between 95 and 125. Expression of the SSTR2 on endothelial cells was noted in some TMA cores (Fig. 5B).

### Prostate-specific membrane antigen (PSMA) expression

PSMA expression was evaluable in *n* = 164 cases. Faint epithelial PSMA expression (maximum IRS = 20) was noted in 4% (*n* = 7) of the total investigated tumors (Fig. 5C). The staining was mainly observed in endometrioid carcinoma (*n* = 4) and, to a lesser extent, in high-grade serous (*n* = 2) and carcinosarcoma (*n* = 1) histotypes. In some tissues, endothelial cells displayed PSMA expression (Fig. 5D).

### Fibroblast activation protein (FAP) expression

Tumor cell expression of FAP (tFAP) was evaluated in *n* = 158 cases, where 10% (*n* = 16) of tumors displayed membranous tFAP expression (Fig. 5E). Among these, one case of a clear cell carcinoma showed high tFAP expression (IRS = 225), whereas the other tFAP-expressing cases showed IRS values between 5 and 100 (average IRS = 53). In both endometrioid and clear cell histotypes, tFAP expression was present in 21% of the cases (*n* = 7 and *n* = 6, respectively). The remaining *n* = 3 tFAP-positive tumors were of high-grade (*n* = 1) and low-grade (*n* = 2) serous EOC histotypes.

Stromal expression of FAP (stFAP) was assessed in *n* = 155 tumors. Across all histotypes, stFAP expression was detected in 47% (*n* = 73) of tumors (Fig. 5F). The proportion of stFAP-positive tumors was comparable among high-grade serous, clear cell and carcinosarcoma tumors, ranging from 56% to 63% of the respective cases (Table 3). In contrast, fewer stFAP-positive cases were observed in low-grade serous and endometrioid histotypes, ranging from 29% to 33%, while none of the mucinous tumors exhibited stFAP expression.

Across all evaluated histotypes, 30% (*n* = 46) of the tumors were classified as stFAP-high and 17% (*n* = 27) as stFAP-low. Beside the rare mixed EOC, which displayed stFAP-high in 100% (*n* = 2) of tumors, stFAP-high was observed most prominently in high-grade serous EOC, corresponding to 42% (*n* = 30) of tumors of this histotype, followed by 33% (*n* = 3) of carcinosarcoma and 32% (*n* = 6) in clear cell EOC. None of the low-grade serous EOC classified as stFAP-high, while 29% (*n* = 2) of the tumors expressed stFAP at low levels and 71% were stFAP-negative (*n* = 5).

To explore the potential for targeting stFAP in FRα-low/negative high-grade serous EOC tissues (and conversely targeting FRα in stFAP-low/negative tumors), each TMA core obtained from high-grade serous EOC was classified as “high” or “low/negative” for both markers. In 16% (*n* = 19) of cores, high stFAP expression was observed in the absence of significant FRα expression, whereas 33% (*n* = 39) of cores showed high FRα expression with low or absent stFAP. In 18% (*n* = 21) of cores, high expression of both targets was observed and in 33% (*n* = 39) of cores, both targets were absent (Supplementary Information; Fig. S3).

**Table 3:** Stromal FAP (stFAP) expression in ovarian carcinoma histotypes

Histotype	Total patients	stFAP-high	stFAP-low	stFAP-negative
High-grade serous	72	30 (42%)	11 (15%)	31 (43%)
Low-grade serous	7	0 (0%)	2 (29%)	5 (71%)
Endometrioid	33	5 (15%)	6 (18%)	22 (67%)
Clear cell	19	6 (32%)	6 (32%)	7 (37%)
Carcinosarcoma	9	3 (33%)	2 (22%)	4 (44%)
Mucinous	13	0 (0%)	0 (0%)	13 (100%)
Mixed	2	2 (100%)	0 (0%)	0 (0%)
Total	155	46 (30%)	27 (17%)	82 (53%)

## Discussion

The histotype proportions of EOC in the investigated cohort largely reflected the reported number of EOC patients in Western Europe. Exceptions were, nevertheless, noted with endometrioid tumors, which accounted for 22% of the cases in our cohort compared to the lower reported incidence of ~10% and in high-grade serous carcinomas, which were underrepresented in this study with 48% as compared to ~ 75% in the Western population [32, 33].

In the present study, FRα expression was detected in 80% of EOCs, slightly exceeding the 65% to 72% range reported by others [34, 35]. Inter-study comparisons are, however, limited by methodological differences in scoring systems, employed antibodies and histotype composition of the evaluated cohorts. Using the PS2 + scoring system, 47% (36/76) of the high-grade serous carcinomas and 43% (3/7) of the low-grade serous carcinomas investigated in this study were classified as FRα-high, which was consistent with previous studies which found frequencies of 44–54% in high-grade serous and 25–40% in low-grade serous EOC [36–39]. The frequency of FRα-high cases determined in this study for endometrioid and clear cell carcinomas (29–35%) was higher than what has been reported by other groups (≤ 8%) [37, 38]. Mucinous carcinomas showed minimal FRα expression with only 1/14 FRα-high case, supporting previous conclusions that patients with this histotype are unlikely to benefit from FRα-targeting approaches [37, 38]. This observation was also supported by the statistical analyses showing a significantly reduced likelihood of “FRα-high” cases in mucinous carcinomas compared to high-grade serous EOC. Overall, the results of this study clearly demonstrated the potential relevance of the FRα in imaging or treatment not only of high-grade serous EOC, but also of the less prevalent cases such as low-grade serous carcinoma, endometrioid and clear cell carcinomas.

In the SORAYA Phase III clinical trial investigating mirvetuximab soravtansine in patients with platinum-resistant high-grade serous EOC, 36% of the patients screened with the IHC-based companion diagnostic test FOLR1-2.1 met the criteria of FRα-high [13]. A recent comparison of anti-FRα antibodies showed higher sensitivity of the 26B3.F2 antibody, which was used in this study, as compared to that of FOLR1-2.1 [40]. The specificity of the 26B3.F2 antibody validated on CHO-FRα and CHO-FRβ xenografts tissue sections, along with comparable findings with other studies performed with EOC tumors [36–39], demonstrated its reliability in detecting and assessing FRα expression.

Few studies have reported FRβ expression on tumor cells in various EOC histotypes, which were observed up to 21% of the cases [9, 41]. While the subcellular localization of FRβ expression was not described in these previous studies, only membranous FRβ was considered in our cohort, in which tumor cells were found to be FRβ-negative. This result was similar to the low overall expression reported in the study of de Boer et al., where 98% of patient samples were FRβ-low/negative [41]. The restriction of membranous FRβ expression to stromal cells in our cohort also inferred its potential role as a marker of the tumor microenvironment, particularly of tumor-associated macrophages and activated macrophages involved in inflammatory processes [8, 11]. Nevertheless, given the more prominent and frequent expression of FRα compared to FRβ in EOC, the β-isoform appears to have limited value as a theragnostic target. Consequently, the specific targeting of the FRα, as is the case for the antibody mirvetuximab soravtansine, represents a more promising strategy also in view of the use of folate-based radiopharmaceuticals.

Folate radioconjugates based on folic acid, which is commonly used as targeting agent due to its high affinity for FRs in general, have demonstrated promising results for the detection of FRα-positive tumors in clinical PET and SPECT imaging studies [16, 42–44]. Pronounced uptake was, however, also found in some healthy tissues including the bone marrow (oral communication), which is highly unfavorable for therapy using folate radioconjugates as this can lead to critical bone marrow toxicity. The accumulation of folic acid-based radioconjugates in the bone marrow was most probably a result of the binding of folic acid to FRβ-expressing bone marrow cells [7]. In addition, the risk of FRβ-mediated false-positive findings was demonstrated in a previous study where lymph node uptake of a folate radioconjugate was attributed to FRβ-positive immune cells rather than to FRα-positive metastatic lesions [43]. This situation emphasizes the need for the development of FRα-selective radiopharmaceuticals in view of radionuclide therapy. Epithelial SSTR2 and PSMA expression was evident in only a small fraction of the EOC tumors with overall low to moderate expression levels, similar to previous studies [45–47]. Currently approved radiopharmaceuticals targeting the SSTR2 and PSMA, therefore, find limited utility and novel radiopharmaceuticals are needed for radionuclide therapy of EOC. In our cohort, epithelial FAP was observed in only 10% of tumors, which was slightly lower than what has been previously reported [47]. In contrast, the presence of FAP in the tumor stroma was frequently observed throughout the cohort, particularly in high-grade serous EOC with 42% of stFAP-high cases. This is in line with previously reported rates of 36–61%, further underscoring the relevance of stFAP in EOC [24, 47]. Multiple FAP-directed imaging agents have demonstrated high tumor-to-background ratios of accumulated activity and excellent diagnostic performance, also for the imaging of ovarian cancer [25, 48, 49]. The therapeutic use of FAP-targeted radiopharmaceuticals is, however, still in its early stage, mainly due to the lack of tumor retention and resultant low dose delivery [50, 51].

Notably, 49% of cores obtained from high-grade serous EOC cases displayed high expression of either FRα or stFAP whereas both targets were highly present in 18% of cores. These data encourage the development of radiopharmaceuticals targeting the FRα or FAP to enable a broader application of radionuclide therapy to high-grade serous EOC where only FRα or FAP will be present in the tumor or the microenvironment.

Limitations of this study refer to the retrospective nature and the random sampling areas of the tissue cores for the TMAs, which may not fully capture the spatial heterogeneity of target expression. Furthermore, the study was performed on tumor samples obtained from treatment-naïve patients, which may not fully reflect the tumor biology of patients with advanced stage disease and a treatment history as they would present for radionuclide therapy. The observed high number of FRα-high cases in endometrioid and clear cell carcinomas warrants confirmation in larger patient groups of these histotypes. Finally, the presence of tumor-associated FRα should be confirmed on fresh tumor tissue samples using the methodology of in vitro autoradiography to unambiguously confirm functional FRs that bind folate-based radiopharmaceuticals.

## Conclusion

FRα and stFAP are frequently expressed at high levels in EOC, particularly in high-grade and low-grade serous carcinomas, but also in less common histotypes. In contrast, the expression levels of PSMA and SSTR2, for which clinically approved radiopharmaceuticals exist, were limited in the investigated cohort of EOCs. These findings support the development of FRα- and FAP-directed theragnostic approaches and their utility in clinical management of EOC.

## Supplementary Information

Supplementary Material 1.

## Data Availability

The data that support the findings of this study are available on reasonable request from the corresponding author. The data are not publicly available due to ethical restrictions.
