# Supplementary material for: Evaluation of folate receptor-alpha and other surface markers as potential targets for radionuclide therapy of ovarian cancer
Source: EJNMMI Res. 2025 Dec 29;15:144. doi: 10.1186/s13550-025-01345-0 (PMC12748496; doi:10.1186/s13550-025-01345-0)
Supplement: Supplementary file 1 — Supplementary Material 1. [file 13550_2025_1345_MOESM1_ESM.docx]

Supplementary Information

**Evaluation of folate receptor-alpha and other surface markers as potential targets for radionuclide therapy of ovarian cancer**

Benjamin D. Hunkeler^1^, Jakob Heimer^2^, Ana Katrina Mapanao^1^, Matthias Choschzick^3^, Cristina Müller^1,4^, Niels J. Rupp^3,5*^

^1^Center for Radiopharmaceutical Sciences, PSI Center for Life Sciences, Villigen-PSI, Switzerland

^2^ Digital Trial Innovation Platform, ETH Zurich, Zurich, Switzerland

^3^Department of Pathology and Molecular Pathology, University Hospital Zurich, Zurich, Switzerland

^4^Department of Chemistry and Applied Biosciences, ETH Zurich, Zurich, Switzerland

^5^Faculty of Medicine, University of Zurich, Zurich, Switzerland

*Corresponding author:

Niels J. Rupp, Prof. Dr. med.

Department of Pathology and Molecular Pathology

University Hospital Zurich

Schmelzbergstr. 12

CH-8091 Zurich

niels.rupp@usz.ch

**1. Growing of xenografts in mice**

***Purpose:*** Xenografts based on cells with known expression of the folate receptor-α (FRα), folate receptor-β (FRβ), somatostatin receptor-2 (SSTR2), prostate-specific membrane antigen (PSMA) and fibroblast activation protein (FAP) were grown in mice and used to validate the specific binding of the antibodies employed for immunohistochemical (IHC) detection.

***Methods:*** All applicable international, national and institutional guidelines for the care and use of laboratory animals were adhered to. Animal experiments were conducted in compliance with the Swiss animal welfare regulations. The studies were approved by the Cantonal Committee for Animal Experimentation, with authorization from the relevant cantonal authorities (License No. 75721 and 75668). Mice were purchased from Charles River Laboratories (Sulzfeld, Germany). Upon arrival at PSI, they were acclimatized for at least one week prior to cell inoculation.

***CHO-FRα/CHO-FRβ.*** Five-to-six week-old severe combined immunodeficient (SCID) CB17 female mice (CB17/lcr-*Prkdc^scid^*/lcrlcoCrl) were subcutaneously injected with 5 × 10^6^ Chinese hamster ovary (CHO) cells transfected with either FRα (CHO-FRα, RT16 cells) or FRβ (CHO-FRβ, D4 cells) in 100 µL PBS as previously reported [1]. These cell lines were kindly provided by Prof. Larry H. Matherly (Wayne State University, Detroit, USA) [2]. Tumor xenografts were collected after approximately 8‒10 days.

***BON-SSTR2/BON.*** Five-to-six week-old female CD1 nude mice (Crl:CD1-Foxn^nu^) were subcutaneously injected with 7 × 10^6^ BON-SSTR2 or 5 × 10^6^ BON cells in 100 µL PBS. BON and the SSTR2-transfected BON-SSTR2 cells are human pancreatic neuroendocrine tumor cell lines kindly provided by Dr. Carsten Grötzinger (Charité–Universitätsmedizin, Berlin, Germany). Tumor xenografts were collected after approximately two weeks.

***PC-3 PIP/PC-3 flu.*** Five-to-six week-old BALB/c nude female mice (Foxn1^nu^/Crl) were subcutaneously injected with 6 × 10^6^ PSMA-positive PC-3 PIP or 5 × 10^6^ PSMA-negative PC-3 flu cells in 100 µL Hank’s balanced salt solution as previously reported [3]. PC-3 PIP and PC-3 flu cells are subclones of the human prostate cancer cell line PC-3 and were kindly provided by Prof. Dr. Martin Pomper (Johns Hopkins University School of Medicine, Baltimore, USA). Tumor xenografts were collected after approximately two weeks.

***HT1080-FAP/HT1080.*** Five-to-six week-old female CD1 nude mice were subcutaneously injected with 5 × 10^6^ cells of the FAP-transfected human fibrosarcoma cell line HT1080-FAP or 3 × 10^6^ HT1080 cells in 100 µL PBS. Both cell lines were kindly provided by Prof. Christoph Renner (Hirslanden Clinics, Zurich, Switzerland) [4]. Tumor xenografts were collected after approximately two weeks.

All tumor xenografts were fixed in formalin and embedded in paraffin. The samples were further processed for immunohistochemistry as described below.

***Results:*** The results of the validation are reported in the main article.

**2. Validation of the antibodies on xenograft tissues**

***Purpose:*** Immunohistochemical detection of the FRα, FRβ, SSTR2, PSMA and FAP was performed on paraffin sections of xenografts with known target expression patterns to validate specific binding of the employed antibodies.

***Methods:*** Immunohistochemistry using the respective anti-human antibodies (Table S1) was performed on 2 µm-thick tissue sections cut from formalin-fixed paraffin-embedded xenograft tissues using automated staining platforms according to the protocols provided below. All slides were scanned using a NanoZoomer scanner (Hamamatsu, Shizuoka, Japan) and evaluated using the viewing software NDP.view2 version 2.9.29 (Hamamatsu, Shizuoka, Japan).

***FRα.*** Detection of the FRα was performed using the prediluted (ready-to-use) monoclonal mouse antibody 26B3.F2 (Biocare medical, Pacheco, USA) on a Ventana BenchMark Ultra stainer (Roche Diagnostics, Rotkreuz, Switzerland). Antigen retrieval was carried out with Ventana Protease 1 (Roche Diagnostics, Rotkreuz, Switzerland) for 4 minutes at 37 °C. The primary antibody was incubated for 30 minutes at room temperature and antibody binding visualized using the Ventana OptiView DAB detection kit (Roche Diagnostics, Rotkreuz, Switzerland). Xenografts of CHO-FRα and CHO-FRβ cells were stained to confirm the specific binding of the employed antibody to FRα.

***FRβ.*** FRβ staining was performed on a Leica Bond III stainer (Leica Biosystems, Nussloch, Germany) using the polyclonal rabbit antibody GTX105822 (GeneTex, Irvine, USA) at a dilution of 1:1600 in Primary Antibody Bond Diluent (Leica Biosystems, Nussloch, Germany). Antigen retrieval was performed with Bond H2 buffer (Leica Biosystems, Nussloch, Germany) for 30 minutes at 100 °C. The primary antibody was incubated for 30 minutes at room temperature and detection carried out using the Bond Polymer Refine DAB kit (Leica Biosystems, Nussloch, Germany). Specific binding of the antibody to FRβ was confirmed on xenografts of CHO-FRβ and CHO-FRα cells.

***SSTR2.*** Immunohistochemical detection of SSTR2 was performed using the polyclonal rabbit antibody RBK046-05 (Zytomed Systems, Langenzersdorf, Austria) at a dilution of 1:25 in Primary Antibody Bond Diluent. Slides were processed on the Ventana BenchMark Ultra platform with antigen retrieval using Ventana CC1 buffer (Roche Diagnostics, Rotkreuz, Switzerland) for 80 minutes at 100 °C. The primary antibody was incubated for 60 minutes at room temperature, followed by visualization with the Ventana OptiView DAB detection kit. Specific binding of the antibody was confirmed on xenografts of BON-SSTR2 and BON cells.

***PSMA.*** PSMA staining was conducted using the monoclonal mouse antibody clone 3E6 (DAKO A/S, Glostrup, Denmark) at a dilution of 1:25 in Primary Antibody Bond Diluent. The protocol was performed on the Ventana BenchMark Ultra platform with antigen retrieval using Ventana CC1 buffer for 32 minutes at 100 °C. Primary antibody incubation was performed for 30 minutes at room temperature, and the Bond Polymer Refine DAB kit used for detection. Specific binding of the antibody was confirmed on xenografts of PC-3 PIP and PC-3 flu cells.

***FAP.*** FAP expression was detected using the monoclonal rabbit antibody EPR20021 (Abcam, Cambridge, UK) at a dilution of 1:100 in Primary Antibody Bond Diluent. Slides were stained on a Leica Bond III platform with antigen retrieval using Bond H2 buffer for 30 minutes at 100 °C. The primary antibody was incubated for 30 minutes at room temperature, followed by detection with the Bond Polymer Refine DAB kit. Xenografts of the FAP-transfected HT1080-FAP and wild-type HT1080 cell line were used for antibody validation.

**Table S1** Antibodies used for immunohistochemical detection of the FRα, FRβ, SSTR2, PSMA and FAP

| **Target** | **Host species** | **Clonality** | **Designation** | **Company** | **Dilution for application** |
| --- | --- | --- | --- | --- | --- |
| FRα | mouse | Monoclonal | 26B3.F2 | Biocare medical | Prediluted (ready-to-use) |
| FRβ | rabbit | Polyclonal | GTX105822 | GeneTex | 1:1600 |
| SSTR2 | rabbit | Polyclonal | RBK046-05 | Zytomed Systems | 1:25 |
| PSMA | mouse | Monoclonal | 3E6 | DAKO A/S | 1:25 |
| FAP | rabbit | Monoclonal | EPR20021 | Abcam | 1:100 |

**Results:** The results of the antibody validation are reported in the main article.

**3. Histotype composition of the ovarian carcinoma cohort**

***Purpose:*** The histotype of the tumors represented on the tissue microarrays (TMAs) were evaluated to identify how each histotype was represented in this study and to assess comparability with current concepts of ovarian carcinoma subtypes.

***Methods:*** The histotypes of the evaluated tumors were classified according to the latest World Health Organization (WHO) classification of Female Genital Tumors (5^th^ Edition) [5], with reference to the essential and desirable criteria.

***Results:*** Most of the EOC tumors were classified as high-grade serous subtype. Other histotypes identified were low-grade serous carcinoma, endometrioid carcinoma, clear cell carcinoma, carcinosarcoma, mucinous carcinoma and mixed carcinoma (Table S2).

**Table S2** Diagnosed histotype of the patients in the ovarian carcinoma patient cohort, expressed as total number of patients with a certain histotype and the proportion of the histotypes among all patients in percent

| Diagnosed EOC histotype | **Number of patients**  **(n)** | | **Percentage of patients per cohort**  **(%)** |
| --- | --- | --- | --- |
| High-grade serous | | 86 | 48 |
| Low-grade serous | | 7 | 4 |
| Endometrioid | | 38 | 21 |
| Clear cell | | 22 | 12 |
| Carcinosarcoma | | 9 | 5 |
| Mucinous | | 15 | 8 |
| Mixed | | 2 | 1 |
| Total | | 179 | 100 |

**4. Immunohistochemical staining of ovarian carcinoma tissue specimens**

***Purpose:*** Immunohistochemical detection of FRα, FRβ, SSTR2, PSMA and FAP was performed on EOC tissue samples to assess the expression of these targets.

***Methods:*** Immunohistochemistry was performed on two TMAs containing 358 tissue cores obtained from 179 EOC patients. The tissues were immunohistochemically stained with the corresponding antibodies as indicated in supplementary section 2.

***Results:*** The results of the immunohistochemical detection of FRα, FRβ, SSTR2, PSMA and FAP in EOC tissue specimens are reported in the main manuscript. The extent of FRα expression for each histotype of total 160 tumors is listed in Table S3.

**Table S3** Expression of FRα in ovarian carcinoma histotypes

| Histotype | **Total patients** | **FRα-high** | **FRα -low** | **FRα -negative** |
| --- | --- | --- | --- | --- |
| High-grade serous | 76 | 36 (47%) | 32 (42%) | 8 (11%) |
| Low-grade serous | 7 | 3 (43%) | 4 (57%) | 0 (0%) |
| Endometrioid | 35 | 10 (29%) | 18 (51%) | 7 (20%) |
| Clear cell | 20 | 7 (35%) | 11 (55%) | 2 (10%) |
| Carcinosarcoma | 8 | 2 (25%) | 2 (25%) | 4 (50%) |
| Mucinous | 14 | 1 (7%) | 2 (14%) | 11 (79%) |
| Total | 160 | 59 (37%) | 69 (43%) | 32 (20%) |

**5. Immunohistochemical detection of FRα on whole slide ovarian carcinoma specimens**

***Purpose:*** Whole slide sections of total n = 4 tumors which showed epithelial FRα expression in TMA cores were immunohistochemically stained in addition to confirm the representability of the TMA cores of the tumor.

***Methods:*** Immunohistochemistry was performed on whole slide tumor specimens of n = 1 low-grade serous carcinoma, n = 2 high-grade serous carcinomas and n = 1 endometrioid carcinoma and using a monoclonal anti-FRα antibody as described in above.

***Results:*** IHC staining of the whole slide sections showed FRα expression patterns concordant to the expression observed on the TMA cores (Fig. S1).

**
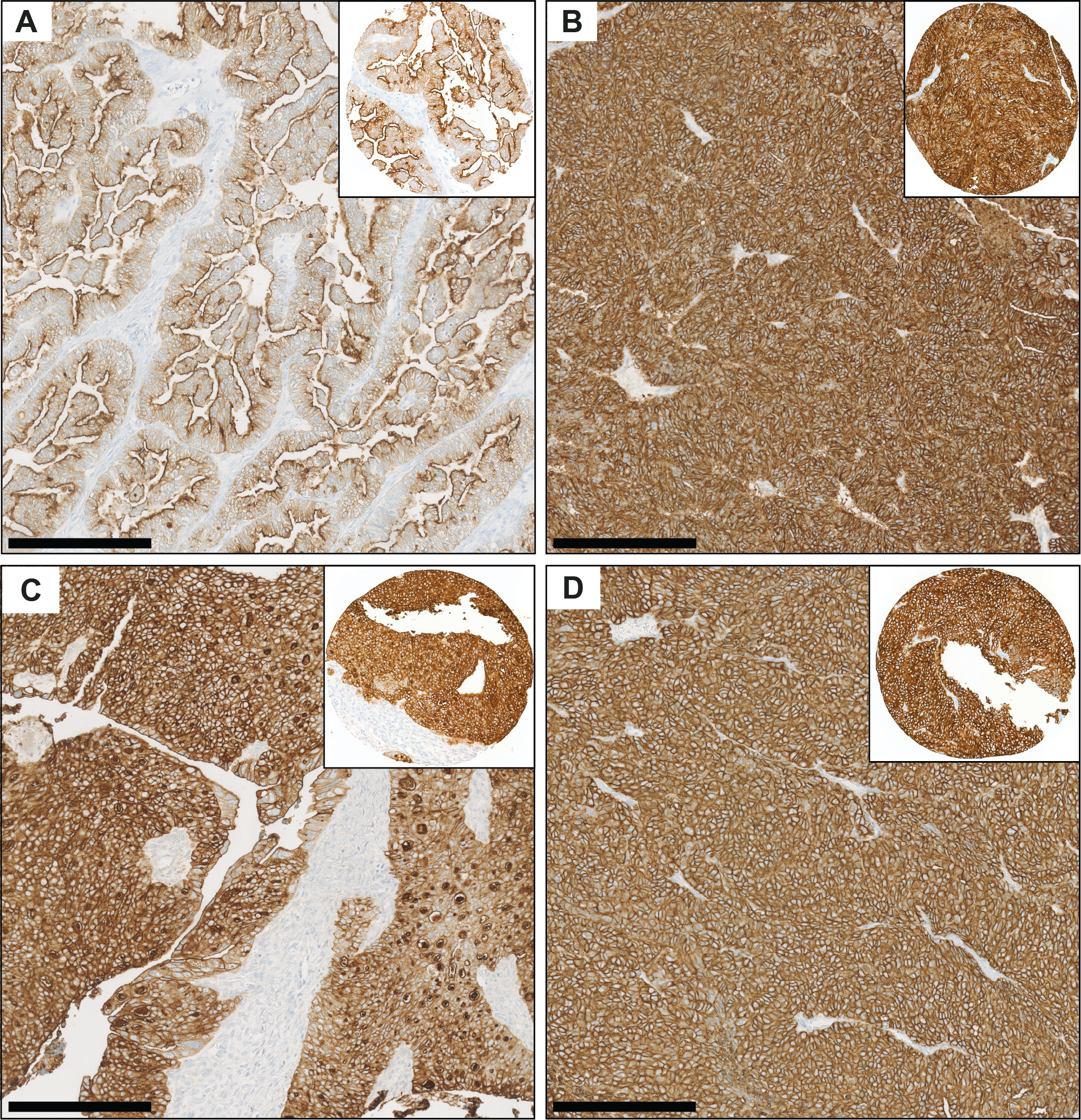
**

**Fig. S1** **A-D** Representative images of whole-slide ovarian carcinoma specimens of various histotypes, immunohistochemically stained for FRα, and the corresponding TMA cores. **A** Low-grade serous histotype, **B/C** high-grade serous histotype and **D** endometrioid histotype of EOC. (Scale bars: 250 µm)

**6. Immunohistochemical detection of FRβ on whole slide ovarian carcinoma specimens**

***Purpose:*** Whole slide sections of total n = 4 tumors which showed no epithelial FRβ expression in TMA cores were immunohistochemically stained in addition to confirm the representability of the TMA cores of the tumor.

***Methods:*** Immunohistochemistry was performed on whole slide tumor specimens of n = 2 high-grade serous carcinomas, n = 1 endometrioid carcinoma and n = 1 low-grade serous carcinoma using polyclonal anti-FRβ antibodies as described in supplementary section 2.

***Results:*** IHC staining of the whole slide sections confirmed the FRβ expression patterns observed on the TMA cores. Expression of FRβ was restricted to stromal cells while no membranous expression was observed on epithelial cells (Fig. S2).

**Fig. S2** Representative image of a **A** whole-slide ovarian carcinoma specimen immunohistochemically stained for FRβ and representative magnification of **B** stromal cells and **C** tumor cells. (Scale bars: 100 µm)

**7. Correlation of FRα and stFAP expression in cores of high-grade serous EOC**

***Purpose:*** The expression patterns of FRα and FAP in tumor cores obtained from high-grade serous EOC were noted to explore the potential for targeting stFAP in FRα-negative/low EOC tissues, and conversely targeting FRα in stFAP-negative/low tissues.

***Methods:*** Immunohistochemistry was performed on two TMAs containing 358 tissue cores obtained from 179 EOC patients. The tissues were immunohistochemically stained with the corresponding anti-FRα and anti-FAP antibodies as described above. Each high-grade serous EOC TMA core was classified as “high” or “low/negative” for both markers.

***Results:*** The correlation of FRα and stFAP expression in high-grade serous TMA cores are reported in the main manuscript and depicted in Fig. S3.


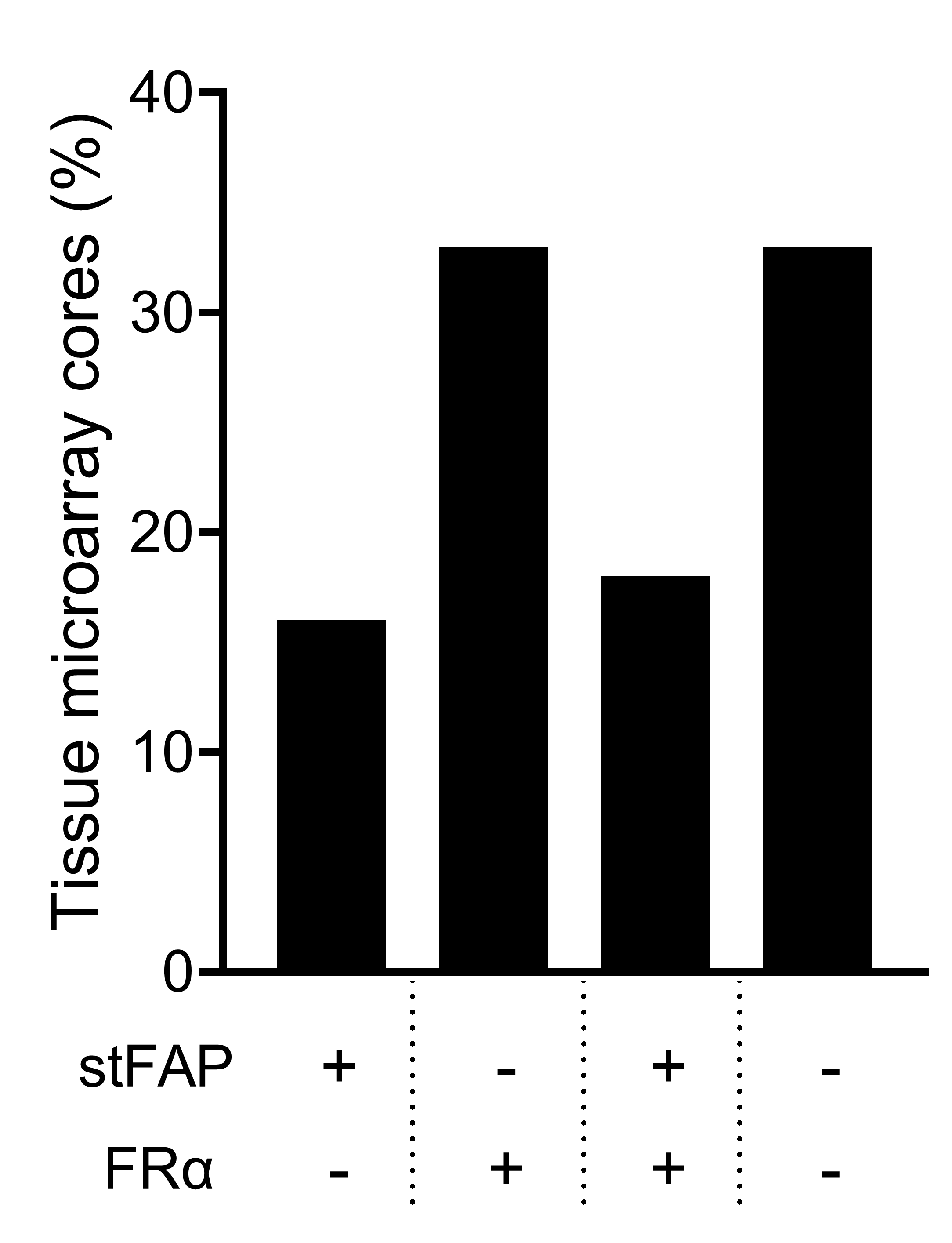


**Fig. S3** The expression status of stFAP and FRα in high-grade serous EOC tissue cores. High expression is designated as (+), whereas low/negative expression is shown as (-)

**References**

1. Guzik P, Fang HY, Deberle LM, Benesova M, Cohrs S, Boss SD, et al. Identification of a PET radiotracer for imaging of the folate receptor-alpha: A potential tool to select patients for targeted tumor therapy. J Nucl Med. 2021;62:1475-81. doi:10.2967/jnumed.120.255760.

2. Deng Y, Wang Y, Cherian C, Hou Z, Buck SA, Matherly LH, et al. Synthesis and discovery of high affinity folate receptor-specific glycinamide ribonucleotide formyltransferase inhibitors with antitumor activity. J Med Chem. 2008;51:5052-63. doi:10.1021/jm8003366.

3. Deberle LM, Benesova M, Umbricht CA, Borgna F, Buchler M, Zhernosekov K, et al. Development of a new class of PSMA radioligands comprising ibuprofen as an albumin-binding entity. Theranostics. 2020;10:1678-93. doi:10.7150/thno.40482.

4. Baird SK, Allan L, Renner C, Scott FE, Scott AM. Fibroblast activation protein increases metastatic potential of fibrosarcoma line HT1080 through upregulation of integrin-mediated signaling pathways. Clin Exp Metastasis. 2015;32:507-16. doi:10.1007/s10585-015-9723-4.

5. McCluggage WG, Singh N, Gilks CB. Key changes to the World Health Organization (WHO) classification of female genital tumours introduced in the 5^th^ edition (2020). Histopathology. 2022;80:762-78. doi:10.1111/his.14609.
